# Supplementary material for: Drought Stress Responses in Arabica Coffee Genotypes: Physiological and Metabolic Insights
Source: Plants (Basel). 2024 Mar 13;13(6):828. doi: 10.3390/plants13060828 (PMC10975139; doi:10.3390/plants13060828)
Supplement: Supplementary file 1 [file plants-13-00828-s001.zip › Supplementary Figures.pptx]

## Slide 1
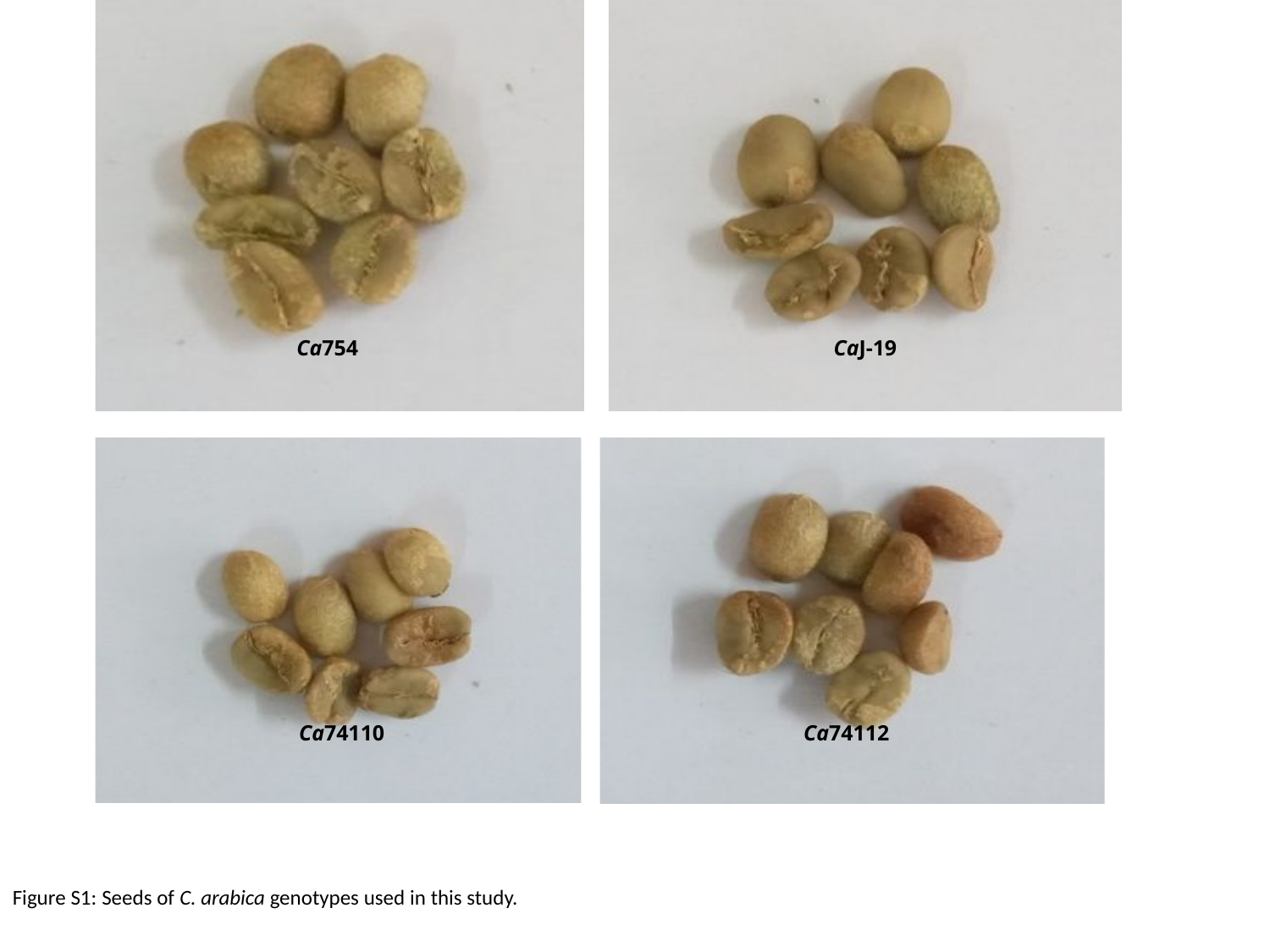

Ca754
CaJ-19
Ca74110
Ca74112
Figure S1: Seeds of C. arabica genotypes used in this study.

## Slide 2
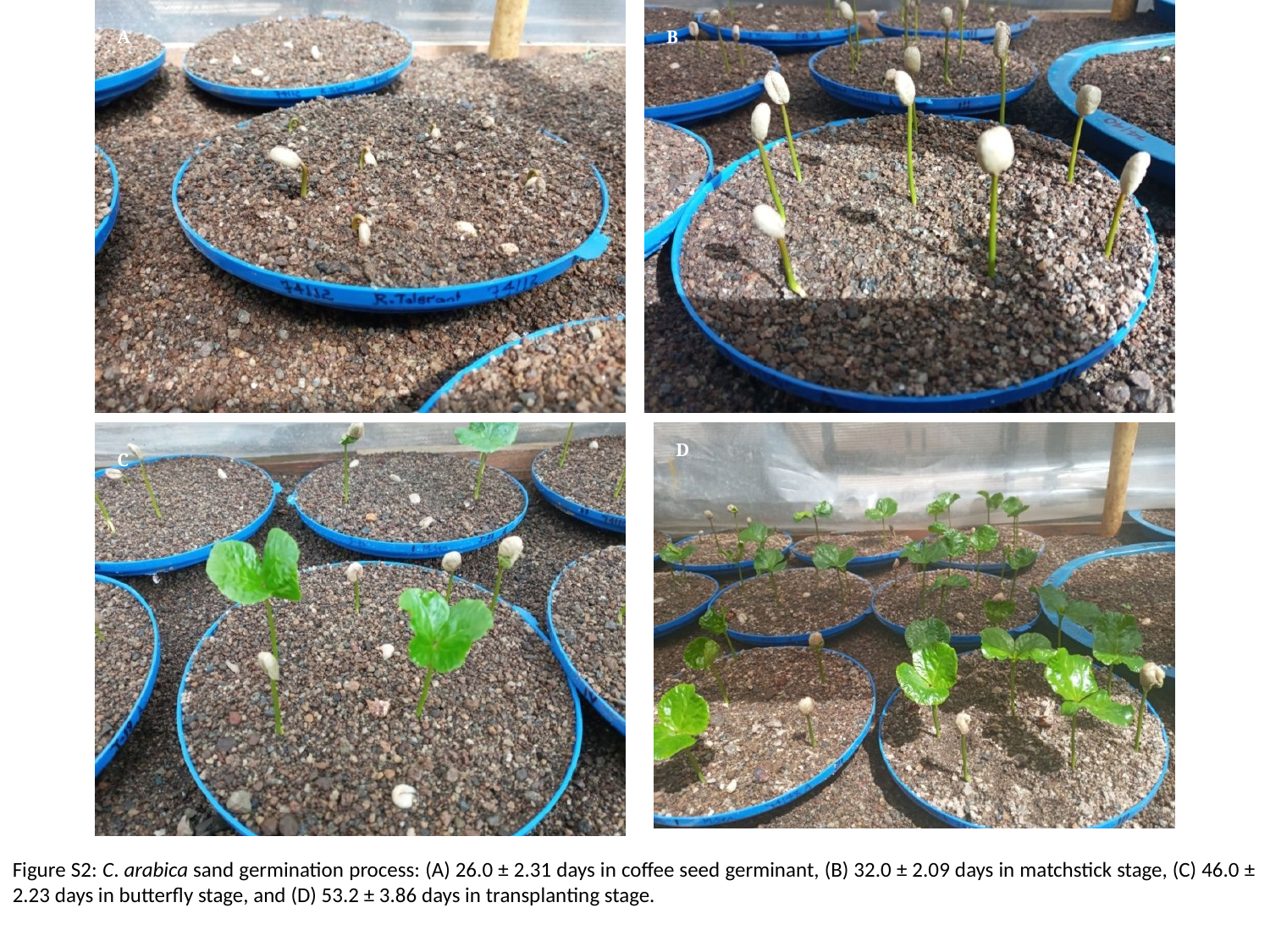

A
B
D
C
Figure S2: C. arabica sand germination process: (A) 26.0 ± 2.31 days in coffee seed germinant, (B) 32.0 ± 2.09 days in matchstick stage, (C) 46.0 ± 2.23 days in butterfly stage, and (D) 53.2 ± 3.86 days in transplanting stage.

## Slide 3
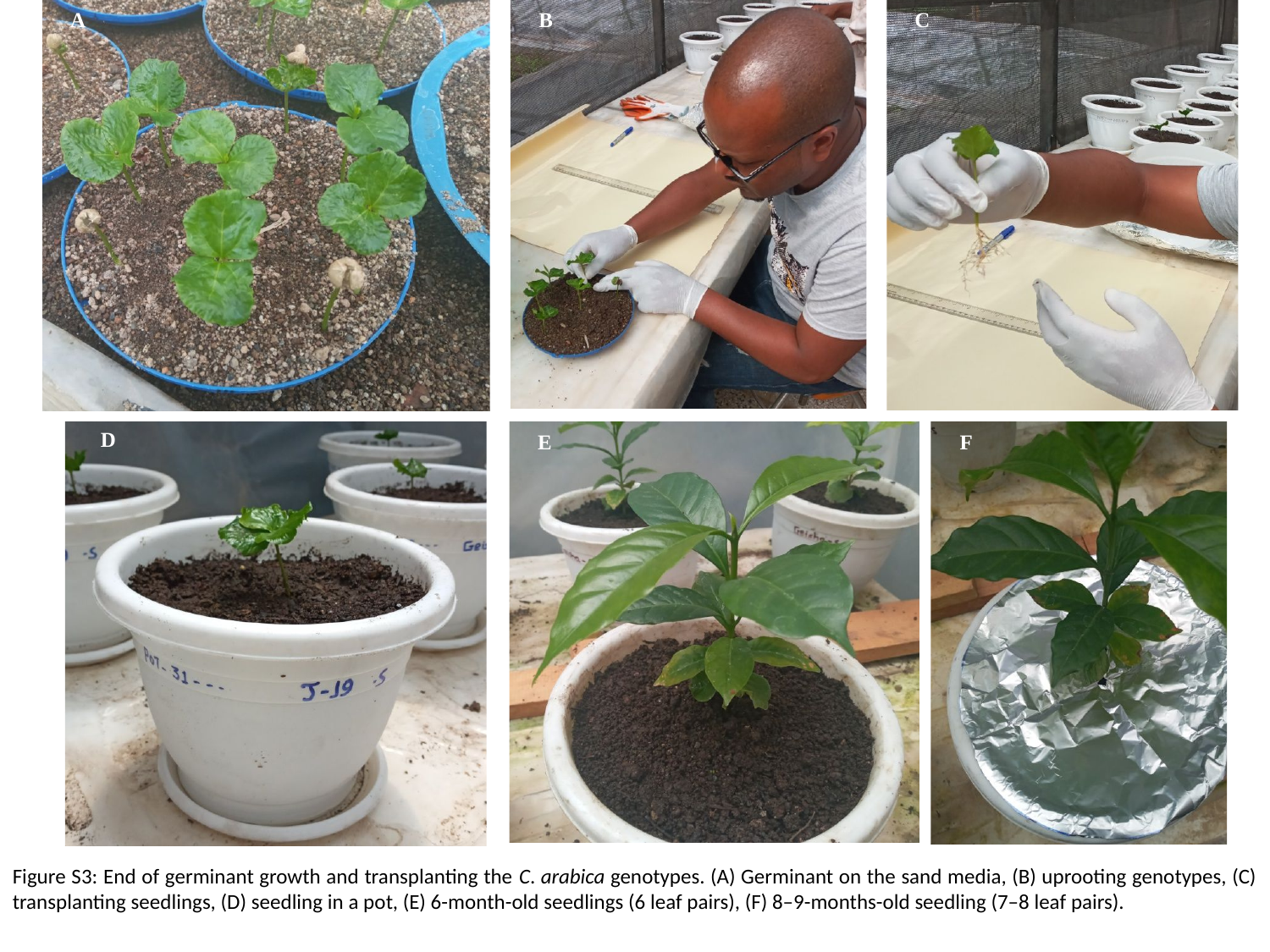

A
B
C
D
E
F
Figure S3: End of germinant growth and transplanting the C. arabica genotypes. (A) Germinant on the sand media, (B) uprooting genotypes, (C) transplanting seedlings, (D) seedling in a pot, (E) 6-month-old seedlings (6 leaf pairs), (F) 8–9-months-old seedling (7–8 leaf pairs).

## Slide 4
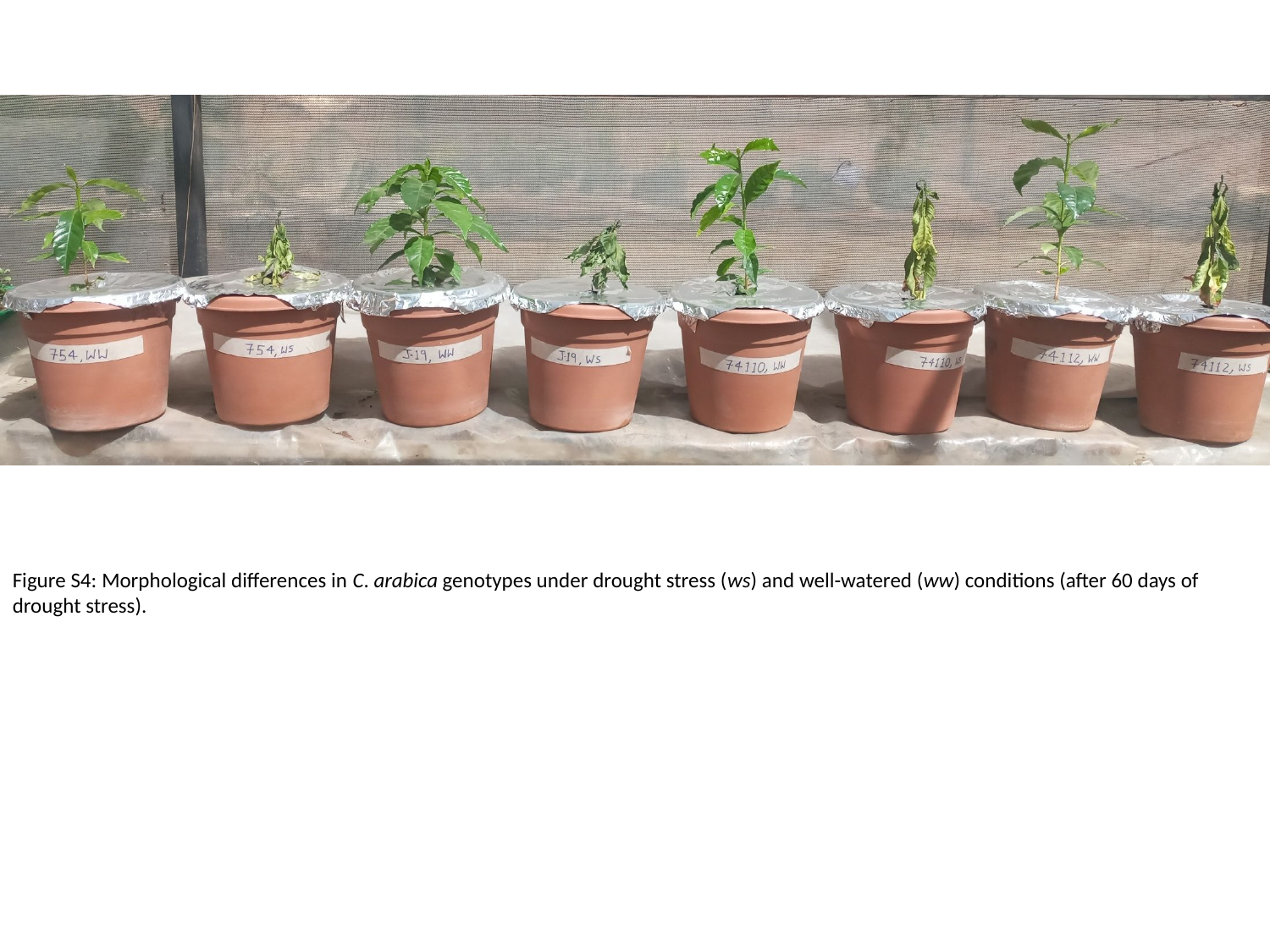

Figure S4: Morphological differences in C. arabica genotypes under drought stress (ws) and well-watered (ww) conditions (after 60 days of drought stress).

## Slide 5
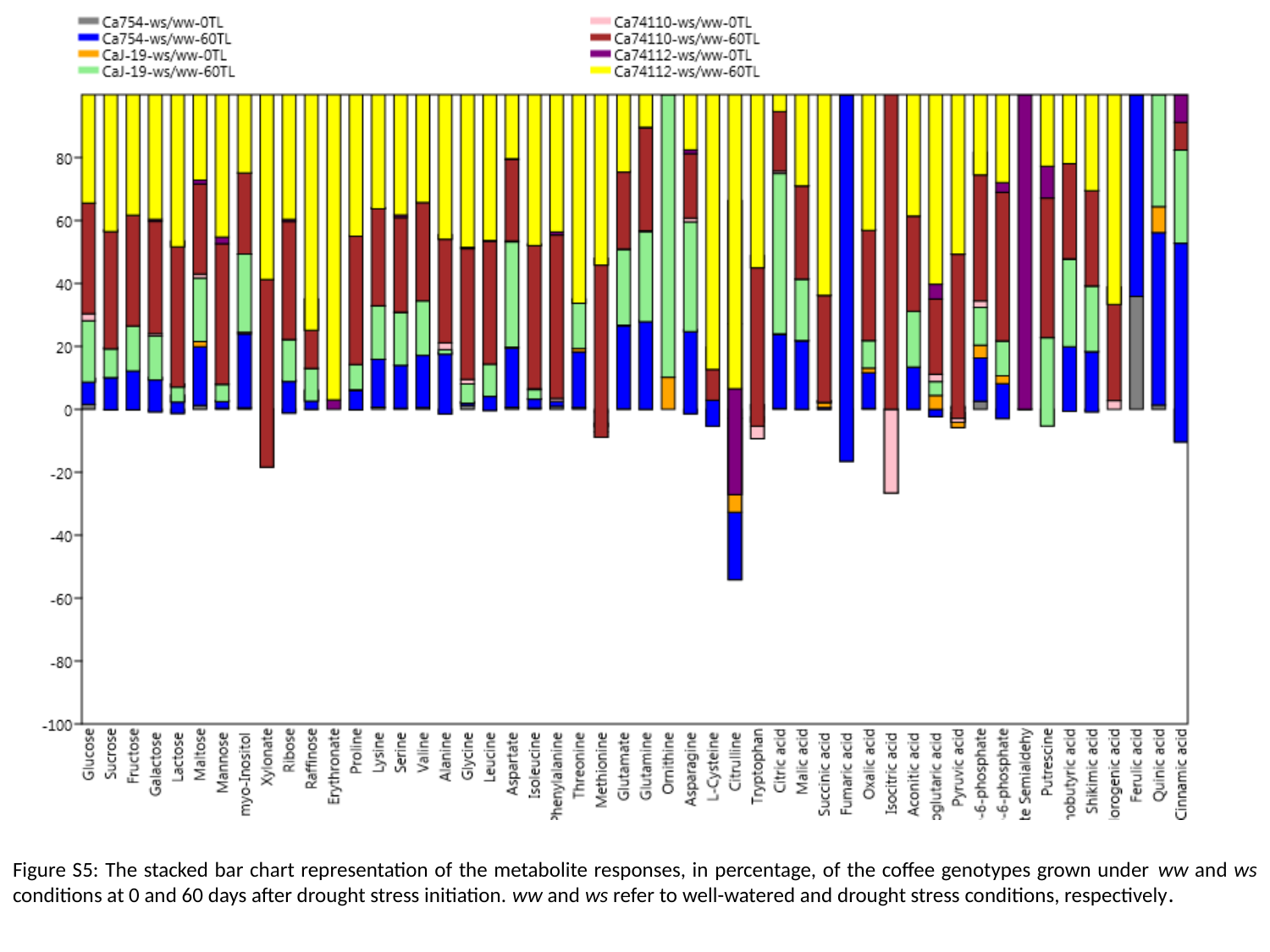

Figure S5: The stacked bar chart representation of the metabolite responses, in percentage, of the coffee genotypes grown under ww and ws conditions at 0 and 60 days after drought stress initiation. ww and ws refer to well-watered and drought stress conditions, respectively.

## Slide 6
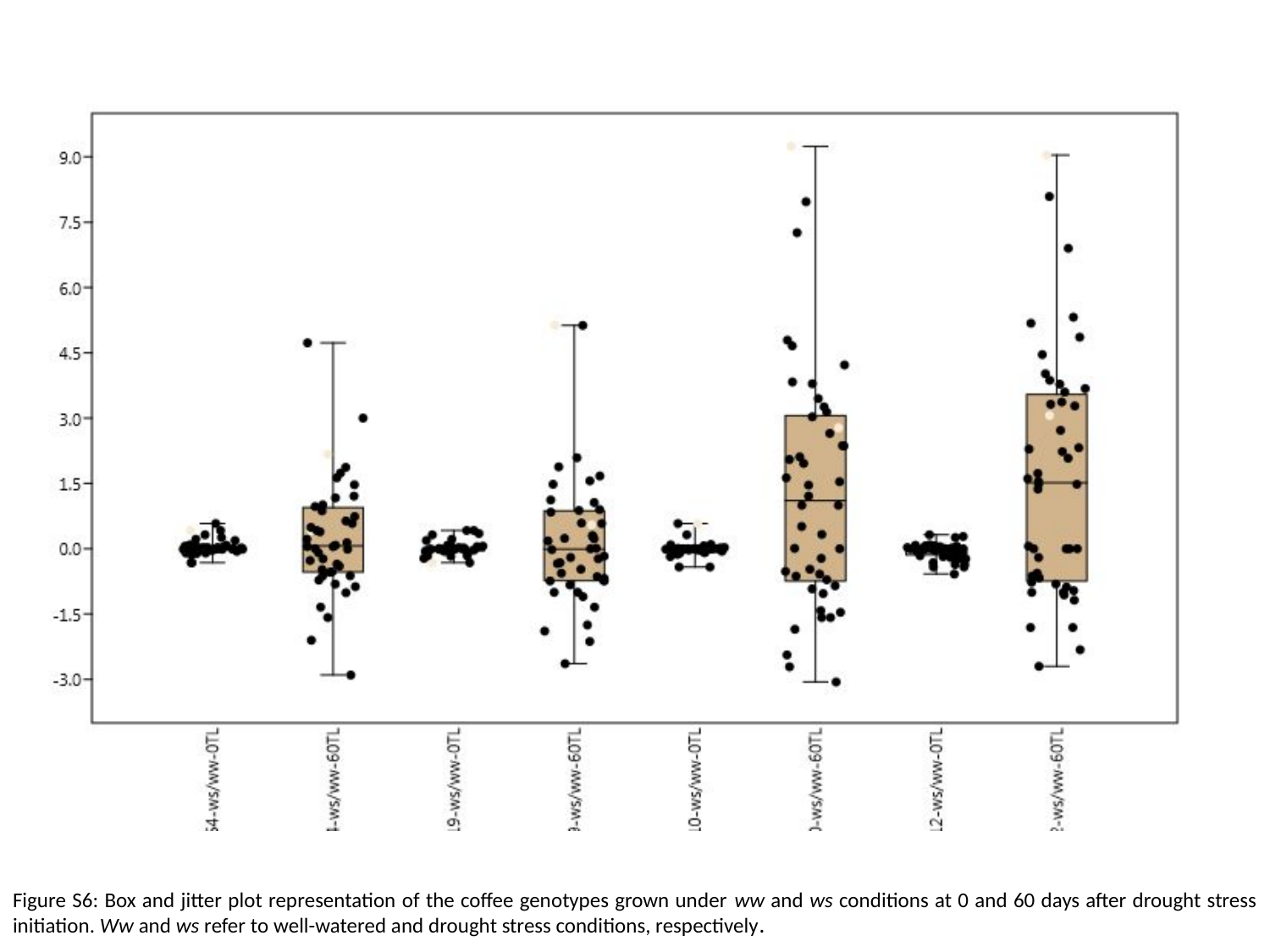

Figure S6: Box and jitter plot representation of the coffee genotypes grown under ww and ws conditions at 0 and 60 days after drought stress initiation. Ww and ws refer to well-watered and drought stress conditions, respectively.

## Slide 7
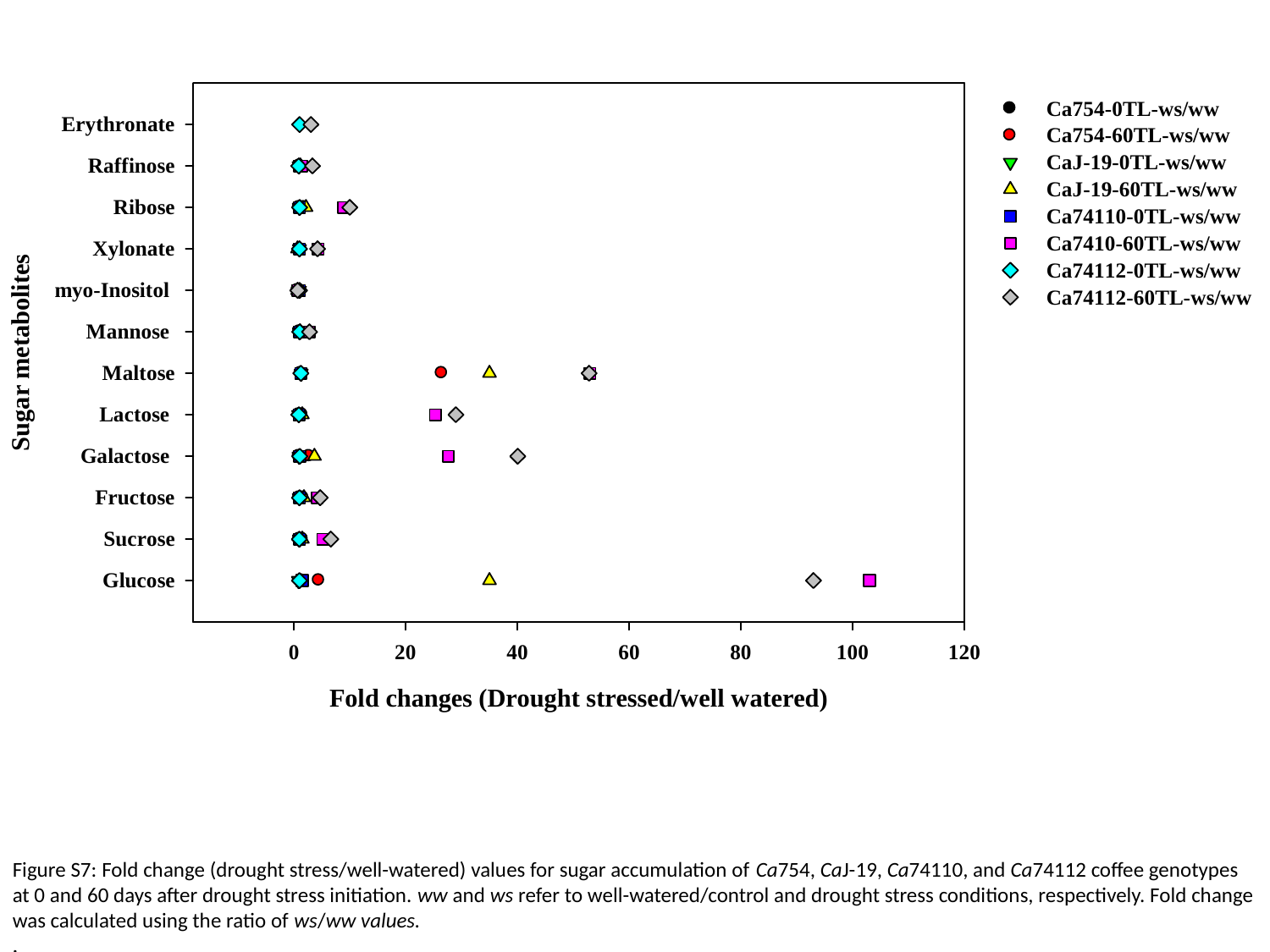

Figure S7: Fold change (drought stress/well-watered) values for sugar accumulation of Ca754, CaJ-19, Ca74110, and Ca74112 coffee genotypes at 0 and 60 days after drought stress initiation. ww and ws refer to well-watered/control and drought stress conditions, respectively. Fold change was calculated using the ratio of ws/ww values.
.

## Slide 8
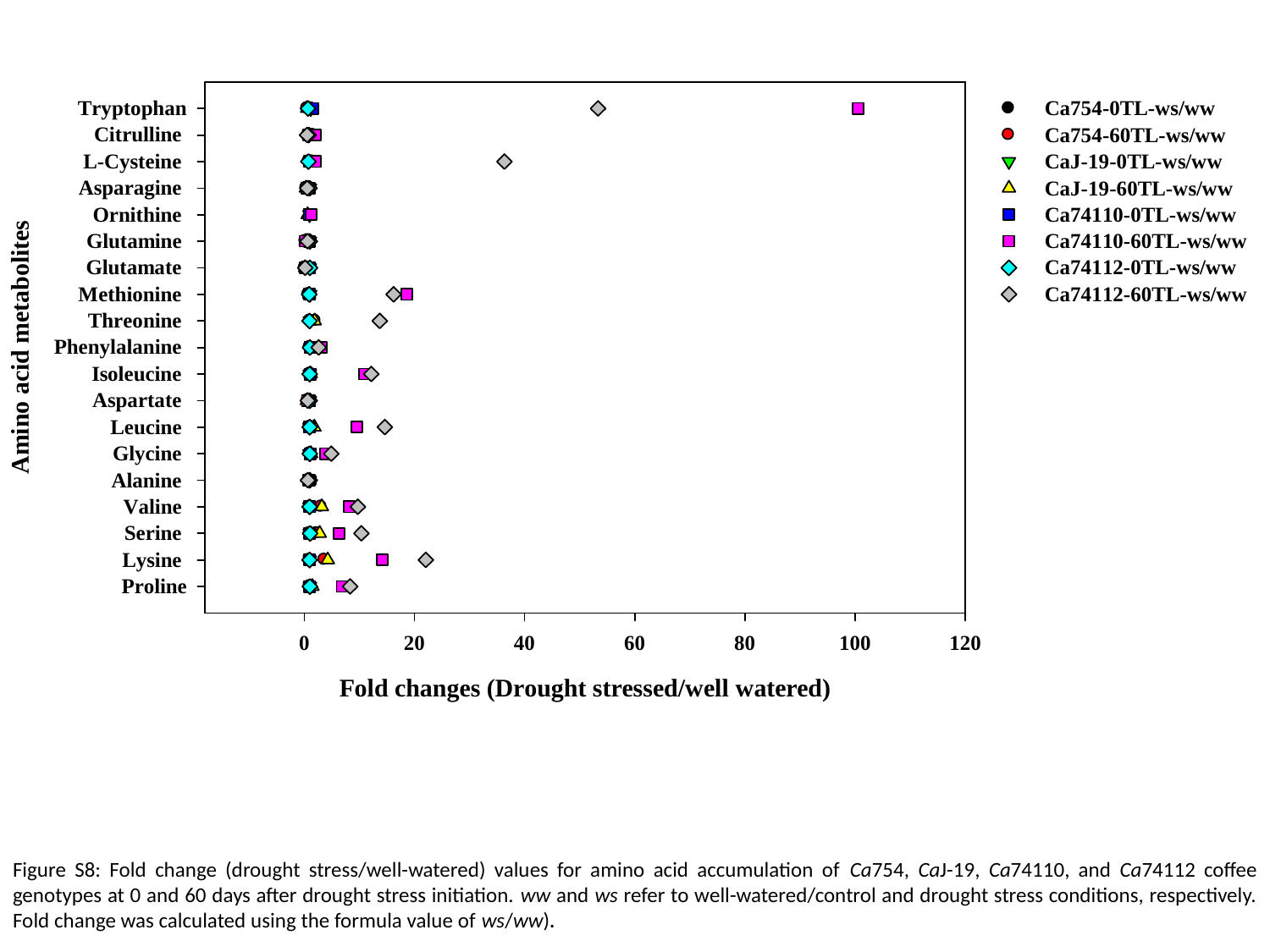

Figure S8: Fold change (drought stress/well-watered) values for amino acid accumulation of Ca754, CaJ-19, Ca74110, and Ca74112 coffee genotypes at 0 and 60 days after drought stress initiation. ww and ws refer to well-watered/control and drought stress conditions, respectively. Fold change was calculated using the formula value of ws/ww).

## Slide 9
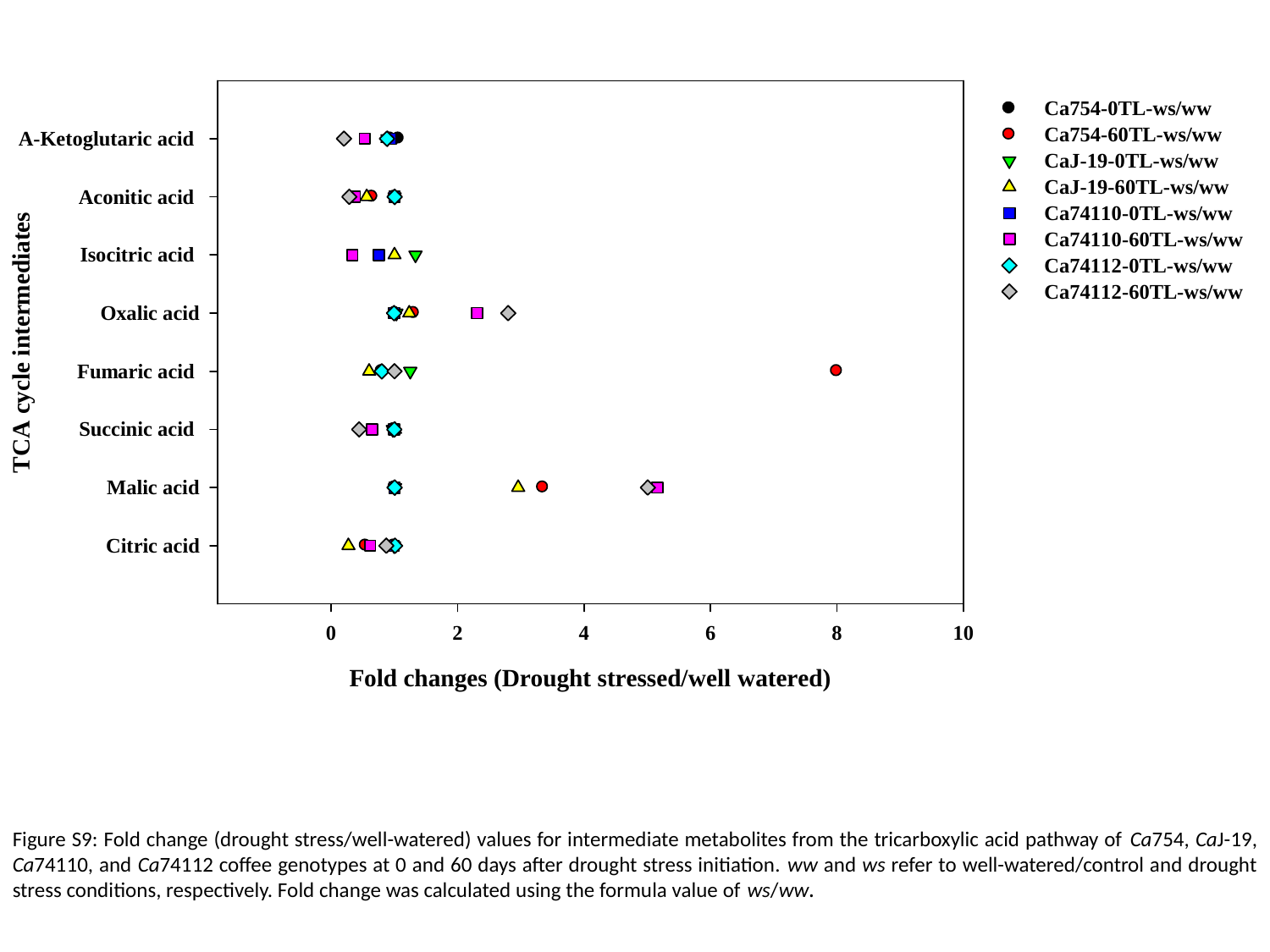

Figure S9: Fold change (drought stress/well-watered) values for intermediate metabolites from the tricarboxylic acid pathway of Ca754, CaJ-19, Ca74110, and Ca74112 coffee genotypes at 0 and 60 days after drought stress initiation. ww and ws refer to well-watered/control and drought stress conditions, respectively. Fold change was calculated using the formula value of ws/ww.

## Slide 10
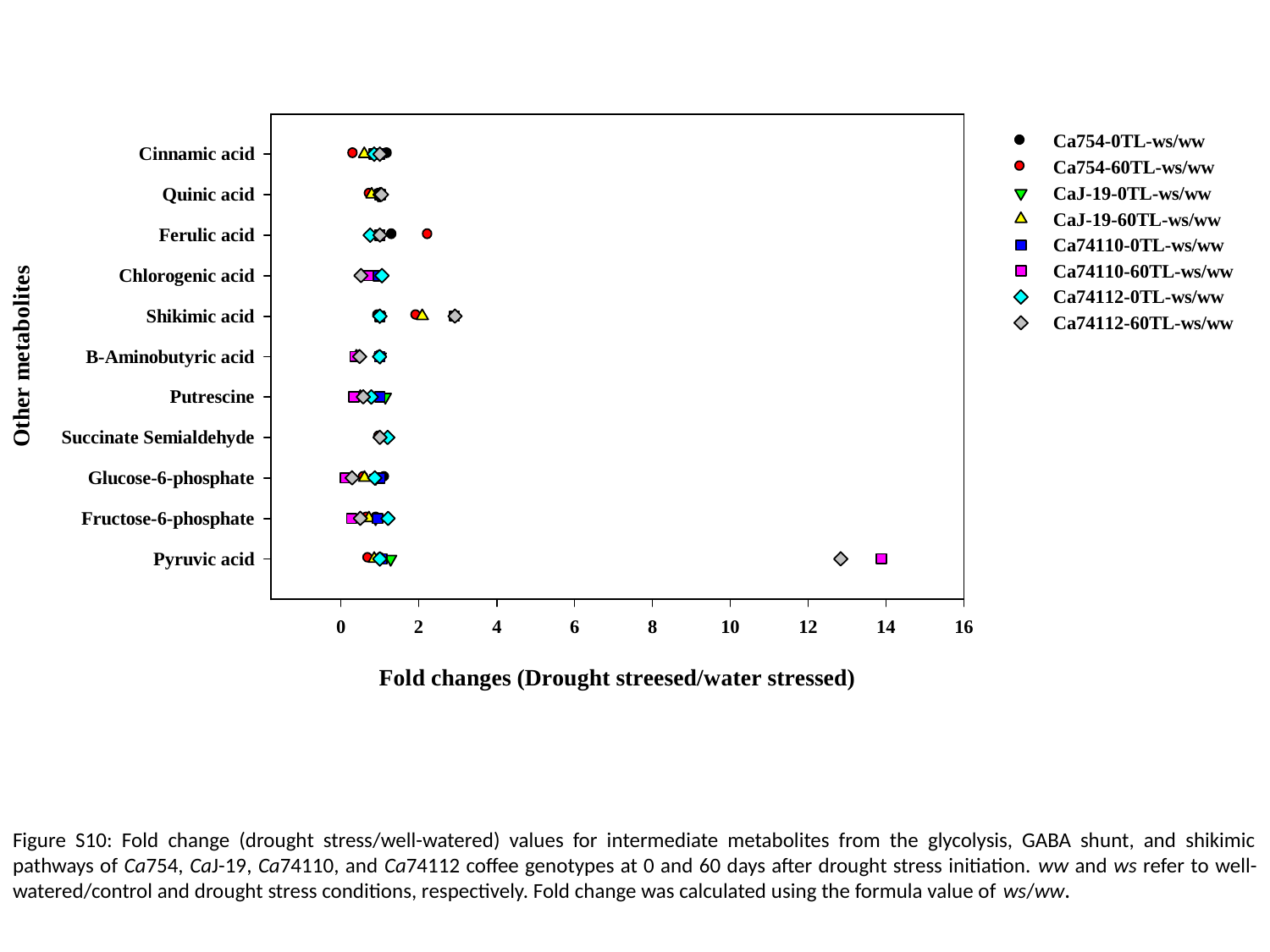

Figure S10: Fold change (drought stress/well-watered) values for intermediate metabolites from the glycolysis, GABA shunt, and shikimic pathways of Ca754, CaJ-19, Ca74110, and Ca74112 coffee genotypes at 0 and 60 days after drought stress initiation. ww and ws refer to well-watered/control and drought stress conditions, respectively. Fold change was calculated using the formula value of ws/ww.

## Slide 11
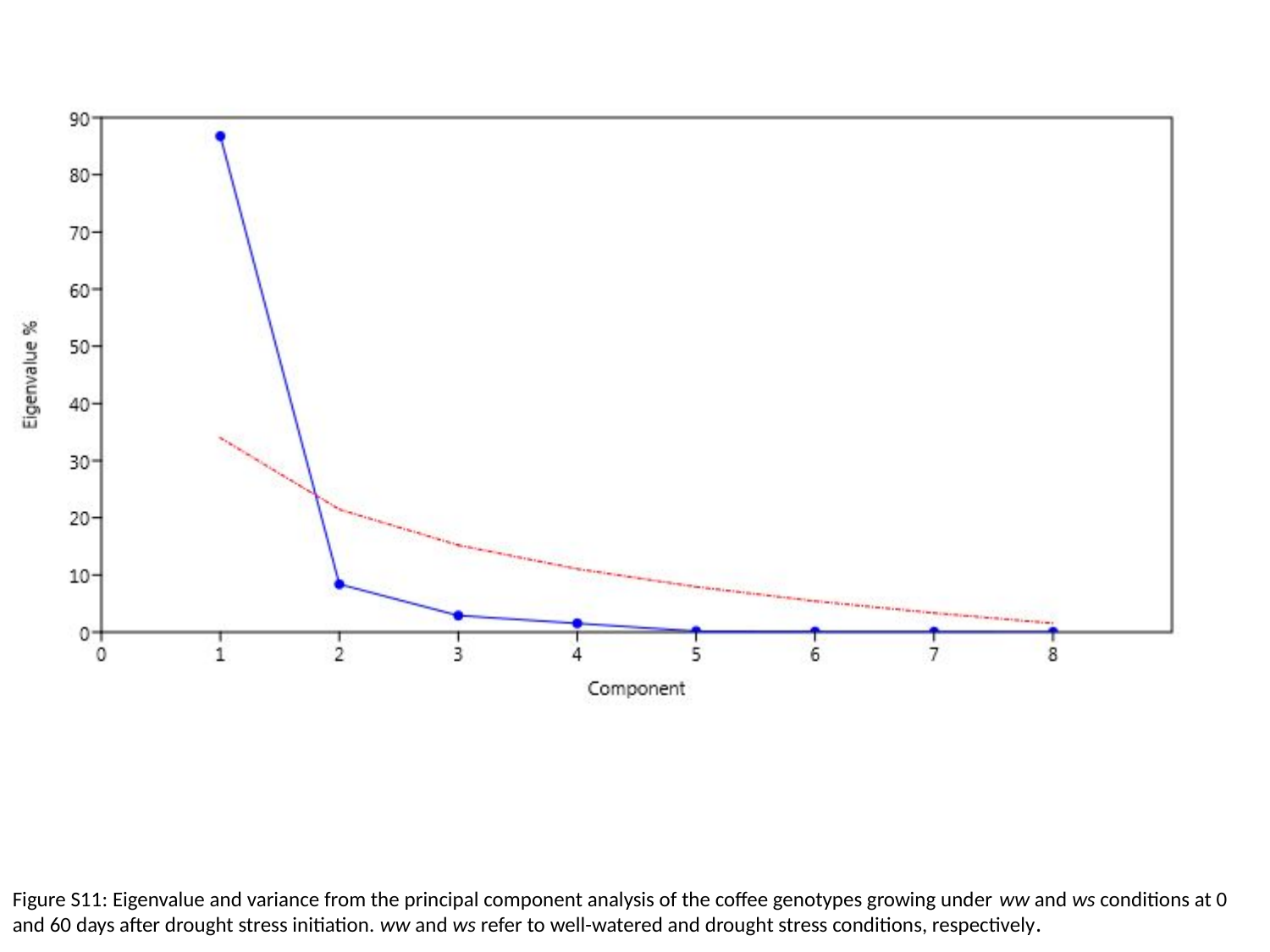

Figure S11: Eigenvalue and variance from the principal component analysis of the coffee genotypes growing under ww and ws conditions at 0 and 60 days after drought stress initiation. ww and ws refer to well-watered and drought stress conditions, respectively.

## Slide 12
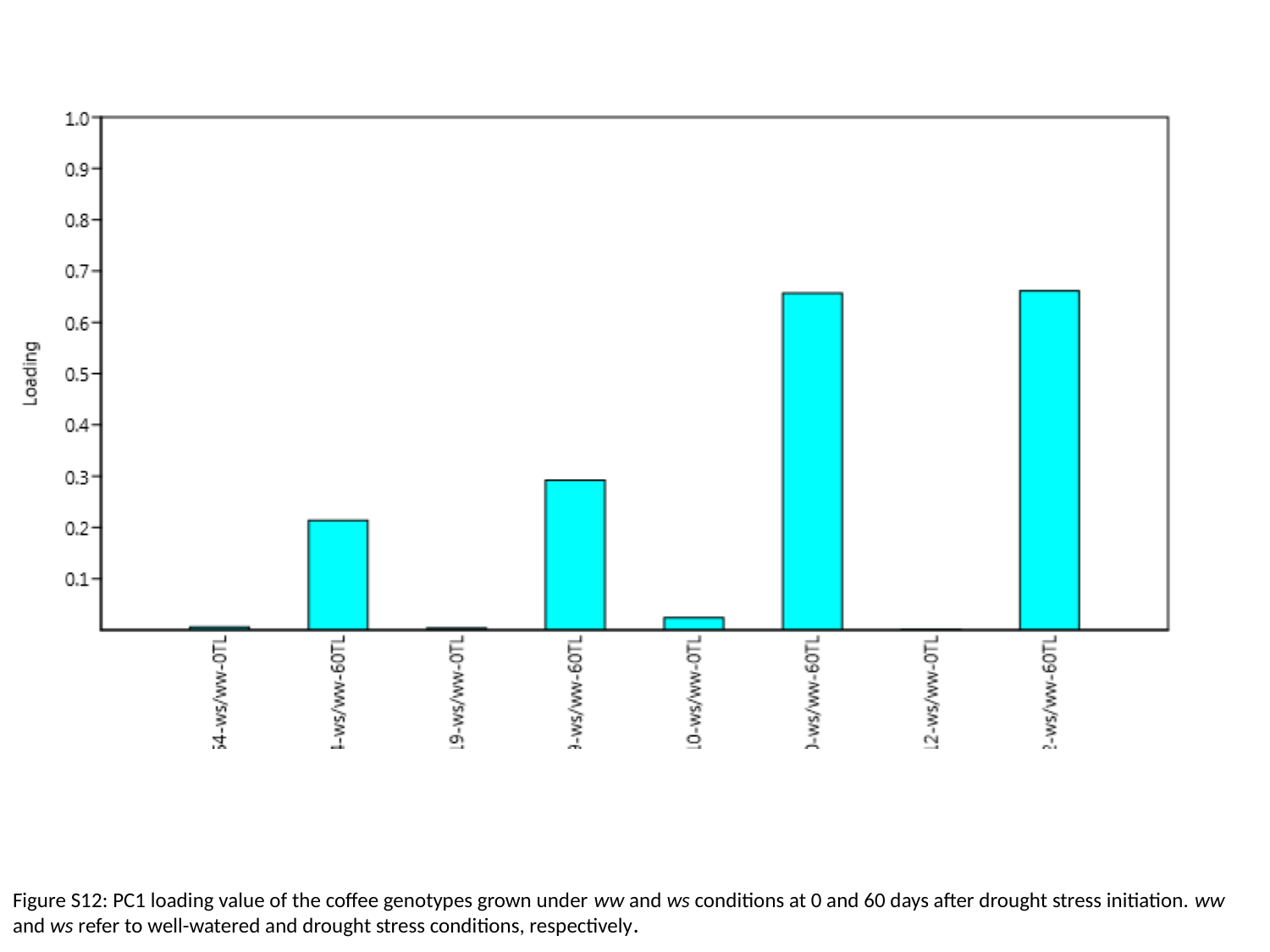

Figure S12: PC1 loading value of the coffee genotypes grown under ww and ws conditions at 0 and 60 days after drought stress initiation. ww and ws refer to well-watered and drought stress conditions, respectively.

## Slide 13
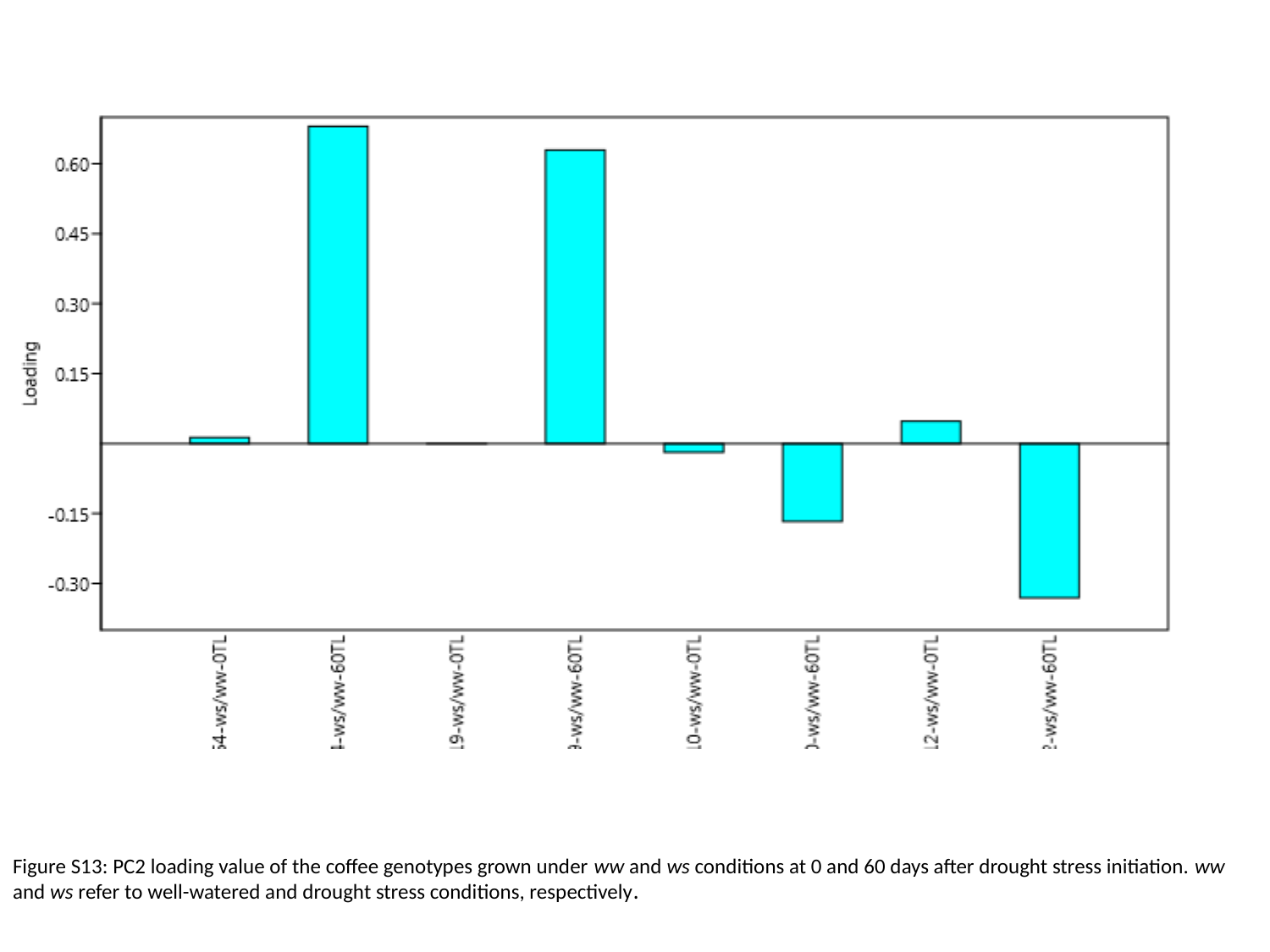

Figure S13: PC2 loading value of the coffee genotypes grown under ww and ws conditions at 0 and 60 days after drought stress initiation. ww and ws refer to well-watered and drought stress conditions, respectively.
